# Editorial: Immunomodulatory Functions of Fibroblast-like Synoviocytes in Joint Inflammation and Destruction during Rheumatoid Arthritis

**DOI:** 10.3389/fimmu.2020.00955

**Published:** 2020-05-20

**Authors:** Hanshi Xu, Song Guo Zheng, David Fox

**Affiliations:** ^1^Department of Rheumatology and Immunology, The First Affiliated Hospital, Sun Yat-sen University, Guangzhou, China; ^2^Department of Internal Medicine, The Ohio State University Wexner Medical Center and College of Medicine, Columbus, OH, United States; ^3^Division of Rheumatology, Clinical Autoimmunity Center of Excellence, University of Michigan Medical School, Ann Arbor, MI, United States

**Keywords:** rheumatoid arthritis, synoviocyte, inflammation, aggressor, immunomodulatory functions

Rheumatoid Arthritis (RA) is a common rheumatic disorder characterized by persistent synovial inflammation and destruction of joints. Fibroblast-like synoviocytes (FLSs) exhibit critical immunomodulatory functions through secretion of inflammatory cytokines and through direct interactions with several synovial-infiltrated immune cell types (1, 2). RA FLSs also display surprisingly aggressive behavior (3), metabolic changes (4, 5), and epigenetic alterations (6, 7). More interestingly, recent studies have identified and described the biological functions of distinct subclasses of RA FLSs, for instance, FAPα^+^THY1^−^ fibroblasts are responsible for bone and cartilage damage, whereas FAPα^+^ THY1^+^ fibroblasts mediate synovial inflammation (8). Another study indicates that THY1^+^HLA-DRA^hi^ fibroblasts contribute to IL-6 expression (9). Increasing evidence suggests that targeting activated FLS may be a novel therapeutic strategy for attenuating RA joint damage (3). This Research Topic brings together original and review articles that explore the immunomodulatory functions of FLS in joint inflammation and destruction in RA.

Bergström et al. conducted a detailed study showing that the autoimmune regulator gene *AIRE* is a cytokine-induced RA risk gene in RA FLS. AIRE is expressed by activated FLS in synovial tissue from RA patients. AIRE does not induce tissue restricted antigens (TRAs) in RA FLS, but augments tumor necrosis factor and interleukin-1β-induced pro-inflammatory response by promoting the transcription of a set of genes associated with systemic autoimmune disease and annotated as interferon-γ regulated genes. These data provide a novel pathological mechanism for association of AIRE with RA.

Yan et al. determined the role of long non-coding RNAs (lncRNAs) HIX003209 in pathogenesis of RA. HIX003209 expression is significantly increased in the peripheral blood mononuclear cells (PBMCs) from RA patients, and is involved in TLR4-mediated inflammation via targeting miR-6089 and thereby releasing regulation of the TLR4/NF-kB pathway in macrophages. Understanding the role of lncRNAs in RA pathogenesis may help to develop innovative therapeutic approaches in the future.

Two groups explored the effects of compounds from phytomedicine on joint damage in RA animal models through their regulation of RA FLS behavior. Wu et al. demonstrated that treatment with Kirenol, a diterpenoid extracted from the Chinese herbal medicine *Siegesbeckiae*, inhibited the migration, invasion, and IL-6 secretion of RA FLS *in vitro*, and improved synovial hyperplasia and cartilage erosion in a collagen-induced arthritis (CIA) mouse model *in vivo*. Du et al. presented that treatment with 3 3-Diindolylmethane (DIM), a main product of the acid-catalyzed oligomerization of indole-3-carbinol from cruciferous vegetables, not only suppressed proliferation, invasion, and inflammatory response of RA-FLS by inhibiting activation of p38, JNK/MAPK, and AKT/mTOR pathways, but also attenuated severity of knee arthritis in f AIA mice. In addition, a cannabinoid receptor 2 (CB2) selective agonist JWH-015 exhibited anti-inflammatory action in human RA FLS by utilizing glucocorticoid receptors. JWH-015 administration inhibited bone destruction in the rat AIA model of RA. These findings may provide an opportunity to develop molecules of similar structure as non-opioid analgesic and bone protective agents in RA.

Diller et al. showed that JAK inhibitors, including tofacitinib, baricitinib, filgotinib, and peficitinib, suppressed the inflammatory responses induced by oncostatin M and by trans-signaling of IL-6 in RA FLS. Indeed, IL-6 and TNF-α play an important role in the pathogenesis of RA (10). Interestingly, only peficitinib reduced the IL-1β-induced inflammatory response and proliferation of RA FLS *in vitro* at concentrations close to reported Cmax values of well-tolerated doses *in vivo*. Peficitinib also significantly inhibited RA FLS migration. This study indicates a possible advantage of peficitinib through targeting of RA FLS, which could result in higher response rates *in vivo* compared to other JAKi.

Finally, the review by de Oliveira et al. discussed glucose metabolism of fibroblast-like synoviocytes as a potential therapeutic target in RA. Key rate-limiting glycolytic enzymes, including hexokinase 2 (HK2) and 6-phosphofructo-2-kinase/fructose-2, 6-biphosphatase (PFKFB) enzymes, play important roles in regulating inflammation and aggressive behavior of RA FLS. Glycolytic inhibitors can inhibit FLS aggressive behavior *in vitro* and attenuate bone and cartilage damage in murine models of RA. Therefore, agents that interfere with certain critical steps of glycolysis may be novel therapeutics, independent of systemic immunosuppression, and suitable for combination therapy in RA.

The articles included in this Research Topic illustrate the complex immunomodulatory functions of FLS in the pathogenesis of RA, and envisage development of potential therapeutic agents for RA by targeting RA FLS. We believe that more detailed understanding of the mechanisms that underlie immunomodulatory functions of FLS will provide novel targets for RA treatment.

## Author Contributions

All authors listed have made a substantial, direct and intellectual contribution to the work, and approved it for publication.

## Conflict of Interest

The authors declare that the research was conducted in the absence of any commercial or financial relationships that could be construed as a potential conflict of interest.
